# 135. Influence of Prescribers on Antibiotic Use among Skilled Nursing Care Residents in 29 U.S. Nursing Homes

**DOI:** 10.1093/ofid/ofab466.337

**Published:** 2021-12-04

**Authors:** Brigid Wilson, Joseph Marek, Robin L Jump, Robin L Jump, Sunah Song

**Affiliations:** 1 Louis Stokes Cleveland VA Medical Center, Cleveland, Ohio; 2 CommuniCare Health Services, Cincinnati, Ohio; 3 Case Western Reserve University, Cleveland, OH; 4 Cleveland Institute for Computational Biology, Clevleand, Ohio

## Abstract

**Background:**

In nursing homes, federal mandates call for more judicious use of antibiotics and antipsychotics. Previous research indicates that practice patterns of nursing home practitioners, rather than resident’s signs and symptoms or overall medical conditions, drive antibiotic use. We hypothesized that nursing home practitioners who prescribe antibiotics more frequently than their peers may display a similar practice pattern for antipsychotics. Here, we examine similarities in prescribing patterns for antibiotics and antipsychotics among practitioners at 29 U.S. nursing homes.

**Methods:**

Prescription data came from 2016 invoices from a pharmacy common to all 29 nursing homes. We defined practitioners as individuals who prescribed ≥1% of systemic medications at a nursing home and excluded practitioners without no prescriptions for anti-hypertensive drugs assuming they were not treating a general nursing home population (i.e. treating hospice or dementia patients). Using anti-hypertensive starts for standardization, we calculated the expected number of starts for both antibiotics and antipsychotics. Using funnel plots with Poisson 99% control limits for the observed-to-expected ratio, we identified practitioners whose use of either class of drugs exceeded these control limits. Practitioners were classified as high, average, or low prescribers for each class of drugs.

**Results:**

We analyzed 129 practitioners who wrote for 113669 systemic medications. For antibiotics, 27 (20%) and 19 (15%) of practitioners were low and high prescribers, respectively. For antipsychotics, 53 (41%) and 14 (11%) were low and high prescribers, respectively (**Figure 1**). Among the low antibiotic prescribers, 59% (16/27) were also low antipsychotic prescribers. Among the high antibiotic prescribers, 21% (4/19) were also high antipsychotic prescribers (**Figure 2**).

Figure 1. (a) Funnel plot for antibiotics

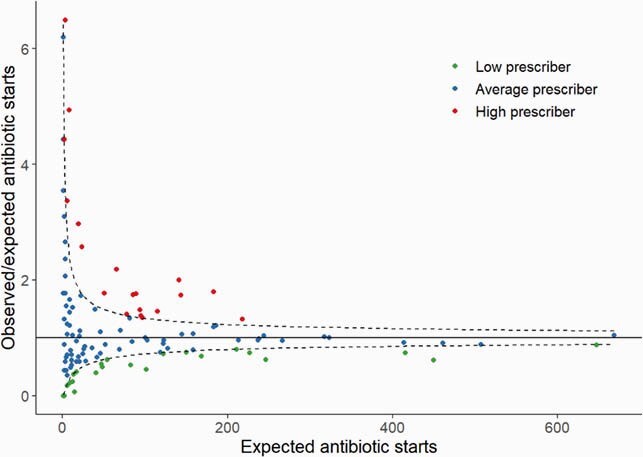

(b) Funnel plot for antipsychotics

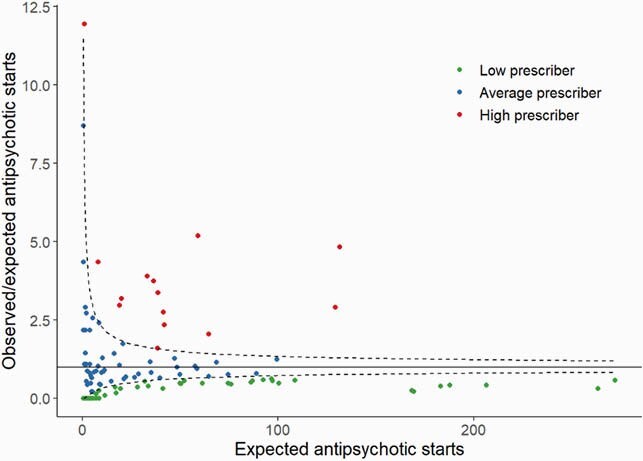

Figure 2. Type of prescriber

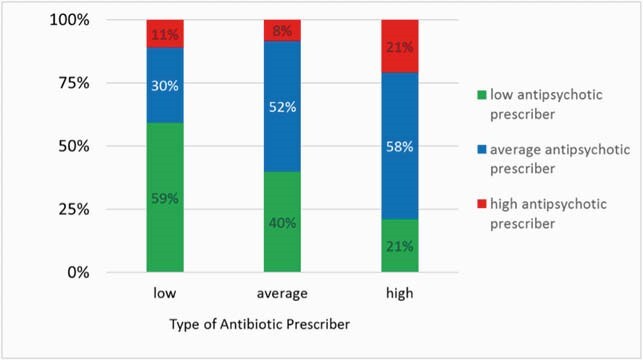

**Conclusion:**

Practitioners who were low prescribers for antibiotics were also likely to be low prescribers for antipsychotics, suggesting judicious use for both classes of medications. Further understanding of the behaviors of these individuals, as well as those who are high prescribers for both classes, has implications for improving antibiotic stewardship practices in nursing homes.

**Disclosures:**

**Robin L. Jump, MD, PhD**, Pfizer (Individual(s) Involved: Self): Consultant

